# Care bundles for women during pregnancy, labour/birth, and postpartum: a scoping review

**DOI:** 10.12688/hrbopenres.13905.1

**Published:** 2024-06-24

**Authors:** Theo Ryan, Tess McGrinder, Valerie Smith

**Affiliations:** 1 Pharmacy and Pharmaceutical Sciences, The University of Dublin Trinity College, Dublin, Leinster, D02, Ireland; 2 Albert Einstein College of Medicine, Montefiore Health System, New York, New York, NY10461, USA; 3 Nursing, Midwifery, and Health Systems, University College Dublin, Dublin, Leinster, D04, Ireland

**Keywords:** Care bundles; Maternity care; Maternal health; Scoping review, Evidence synthesis

## Abstract

**Background:**

Care bundles, introduced in 2001, are described as a set of at least three evidence-based healthcare interventions delivered together in a clinical care episode by all healthcare providers. Although widely implemented in some healthcare areas, care bundle use in maternity care appears relatively recent. To identify the types of care bundles that have been developed, evaluated, or implemented for women during the perinatal period, we undertook a scoping review.

**Methods:**

Joanna Briggs Institute methodological guidance for scoping reviews was used. MEDLINE, CINAHL, Embase, Maternity and Infant Care, and Epistemonikos were searched from January 2000 to October 2023. Records that reported on women during pregnancy and up to six-weeks postpartum as the intended or actual recipients of a care bundle, were included. The concept of interest was development, evaluation, or implementation of a care bundle. The context was maternity care provision, in any setting or geographical location.

**Results:**

The search yielded 147 eligible records of which 69 originated in the USA. Most records were concerned with care bundle evaluation (n=74), and most were published in the last five-years (n=95). Eleven categories of clinical conditions were identified. These were surgical site infection, obstetric haemorrhage, perineal trauma, sepsis, stillbirth, hypertension, safe reduction of caesarean section, enhanced recovery after caesarean, placenta accrete, perinatal anxiety/depression, and ‘other’ which contained 21 records reporting on care bundles for one clinical condition. Few clinical conditions had good overlap of care bundle elements. Systematic reviews based on data from non-randomised studies may be feasible for some clinical conditions.

**Conclusions:**

This scoping review provides comprehensive insight on care bundles in maternity care. Few studies were found that evaluated the effectiveness of these bundles, and many bundles for similar clinical conditions contained diverse elements. A more global approach to care bundle development, evaluation, and implementation in maternity care is recommended.

## Introduction

In national and international guidelines, high-quality, woman-centred maternity care is a priority regardless of healthcare setting or geographical location ^1, 2^. Maternity care spans the period from pregnancy through to labour and birth and postpartum, usually concluding around six-weeks after childbirth ^3^. Care provided during this time is wide-ranging, encompassing physical health surveillance and management, as well as emotional, psychological, and psychosocial assessment and support. Engendering safe maternity care requires the ongoing development, assessment, and improvement of care procedures, and the assimilation of evidence-based practices or interventions into clinical care. Interventions known to improve maternal health outcomes are numerous, for example, the use of uterotonics in preventing postpartum haemorrhage ^4^, prophylactic antibiotics for women undergoing caesarean birth to prevent infection ^5^ and calcium supplementation which reduces the incidence of pre-eclampsia by more than half in all women, compared to no supplementation ^6^. As evidence of effect for interventions emerge, these interventions tend to be implemented in isolation as discrete care practices. Recent care developments, however, are highlighting the use of combining several interventions, which already exist as part of standard care, and collectively implementing these as a ‘care bundle’ ^7^.

### Care bundles

The origin of care bundles dates to 2001 when the Institute for Healthcare Improvement (IHI), a not-for-profit organization in the United States, first described a care bundle as *“a small set of evidence-based interventions for a defined patient segment/population and care setting that, when implemented together, will result in significantly better outcomes than when implemented individually*.” ^7, p.2^ A minimum of three interventions that already exist as part of standard care are generally assumed to comprise this ‘small set’ ^8^. As an example, one of the first care bundles introduced was the IHI ventilator bundle which consists of five intervention elements ( Box 1) ^8^.

**Box 1. IHI Ventilator Care Bundle Elements ^8^**

-   Elevation of the head of the bed to between 30 and 45 degrees

-   Daily sedation vacations and assessment of readiness to extubate

-   Peptic ulcer disease prophylaxis

-   Deep venous thrombosis prophylaxis

-   Daily oral care with chlorhexidine

The premise for care bundle use is predicated on the notion that combining evidence-based interventions together as elements in a bundle which is then *“applied to an individual in one clinical episode”* ^p.2^ by all healthcare providers, every time and in their entirety, then both care quality and care outcomes are improved ^9^. Since 2001, evidence of effect for care bundle use has emerged. For example, a study conducted in Belgium demonstrated that care bundles designed to reduce or prevent ventilator-associated pneumonia (VAP) reduced the rate of VAP by over half in four years, from 28% in 2010 to 11% in 2014 ^10^. Similar positive effects have been noted with a surgical site infection care bundle with a reported reduction in rates from 11% to 4% following implementation ^11^. Although now established in some healthcare areas for some clinical conditions ^10– 12^, the use of care bundles in maternity care appears recent, although gaining momentum. One such example is the obstetric anal sphincter injuries (OASI) care bundle which was developed in 2016 in the United Kingdom (UK) in response to concerns over the rising rates of OASI ^13^. This care bundle, which contains four elements, is designed for use by healthcare providers in caring for a woman’s perineum during childbirth. A stepped wedge cluster trial designed to evaluate the effectiveness of the care bundle in the UK, demonstrated a 20% reduction in the risk of OASI after its introduction ^14^. Further evaluation through an upscaled implementation-effectiveness project is planned ^15^.

Given the probable expansion of care bundles for use in maternity care, and to mitigate the potential for duplication of care bundle development across maternity settings and regions, we sought to determine the scope of care bundle development, evaluation, and implementation in maternity healthcare. A search of systematic review databases and registries revealed only one PROSPERO-registered protocol on care bundles related to maternity care ^16^. The protocol, however, is presented as a rapid review of the literature to 2018 and focuses on the use of care bundles in midwifery care only ^16^. The review thus differs to our scoping review as the participants are midwives and the scope is narrower. In this regard, as far as we are aware, no comprehensive scoping review on the use of care bundles in the broader context of maternity care has been undertaken. For this reason, we sought to ascertain what care bundles have been developed, evaluated, or implemented in caring for pregnant and postpartum women and for what clinical conditions. By undertaking a scoping review the potential for future systematic review conduct on maternity care bundles may also be established.

## Review objectives

The objectives of the scoping review were to:

1. Identify care bundles that have been developed, evaluated, or implemented in caring for women during pregnancy, labour, birth, and up to six-weeks postpartum.
2. Describe the clinical conditions for which these care bundles are being applied.
3. Describe the intervention elements of the identified care bundles, that is, the individual treatments or practices that constitute the bundle.
4. Identify the type of evidence available for these care bundles to determine the need for future systematic reviews.

## Methods

The protocol for this scoping review has been published ^17^ and the review was registered prospectively on Open Science Framework (OSF) ( https://osf.io/hp5vq/). We used the Joanna Briggs Institute (JBI) methodological guidance for scoping reviews in conducting the review ^18^. In reporting the review, we adhere to the Preferred Reporting Items for Systematic Reviews and Meta-Analyses extension for Scoping Reviews (PRISMA-ScR) ^19^ (Supplementary File 1: https://osf.io/hp5vq/).

### Inclusion criteria

The Participants, Concept, Context (PCC) framework ^18^ and types of evidence sources was used to frame the review’s inclusion criteria.

***Participants*.** Records that report on women during pregnancy through to six-weeks postpartum, as the intended or actual recipients of a care bundle, were eligible for inclusion. Women could be primiparous or multiparous, of any age or risk level for perinatal complications, and in receipt of care from a maternity health professional (e.g., obstetrician, family physician, midwife) during the perinatal period.

***Concept*.** The concept of interest was records that reported on the development, evaluation, or implementation of a care bundle. For purposes of the review, a care bundle was defined using the IHI definition; that is, consisting of at least three evidence-informed practices that are packaged together as a ‘bundle’ and implemented consistently by healthcare professionals ^7^. To be eligible for inclusion, the concept had to be explicitly described as a ‘care bundle’ in the record, and not implied. Acknowledging that maternity care involves caring for women as well as the baby, care bundles designed exclusively for neonatal care were excluded. This is because following birth, the neonate is recognised as a separate individual with discrete care needs. This may include care in the neonatal intensive care unit where separate neonatal care bundles are available ^20– 21^. Care bundles which were designed for maternal care but also incorporated an element related to fetal care, for example, intrapartum fetal monitoring, were, however, considered for inclusion in the scoping review.

***Context*.** The contextual setting for the scoping review was maternity care in any environment (hospital, in a primary care or community setting or in the home), in any geographical region or country. Maternity care, in this review, refers to the time of pregnancy through to labour, birth and up to six-weeks postpartum.

***Types of evidence sources*.** As this is a scoping review, no limitations were applied to the types of records that were eligible for inclusion. Controlled before-and-after studies, interrupted time-series studies, case-control studies, analytical or descriptive cross-sectional studies, development/validation studies, randomised and non-randomised clinical trials, and prospective and retrospective cohort studies were of interest. Qualitative studies that explored women’s perspectives of care bundles during maternity care were also considered for inclusion, as were policy or practice guidelines, texts, or opinion papers pertaining to care bundle development, evaluation, or implementation. Records that only described, commented on, or critically reviewed a bundle were ineligible for inclusion and were categorised as *‘wrong concept’*. Similarly, to retain scope within our predefined population of women, records relating to clinicians’ opinions, views or experiences of a maternity care bundle were excluded.

### Search strategy

A comprehensive search strategy was designed and implemented on 17 October 2023. The start date for the search was 01 January 2000 which was chosen based on the 2001 date when care bundles were first introduced by the IHI. Setting this date, we believed, would optimise the likelihood of any identified care bundles meeting the IHI definition. Records that were published, unpublished, planned, or ongoing that met the review’s PCC criteria were considered for inclusion. Language limitations were not applied to the search strategy; however, due to an inability to translate non-English records with complete accuracy, only records published in English were included. By searching all languages, however, we could identify non-English records that might have potentially met the review’s inclusion criteria and note these as a possible source of language bias.

***Information sources*.** The electronic databases of MEDLINE (OVID), CINAHL (EBSCO-host), Embase (EBSCO-host), Maternity and Infant Care (MIDIRS), and Epistemonikos were searched. To inform the search strings, a preliminary search of MEDLINE (OVID) was undertaken using the terms “care bundle” AND “maternity OR pregnancy.” This search retrieved 345 records. The text words contained in the titles and abstracts of these records and the index terms used to describe the records were used to inform the final search strategy for the scoping review. A previous search string for the ‘care bundle’ concept that was developed and used in a systemic review of care bundles for patients with COVID-19 in the intensive care unit (led by author VS) ^22^, informed the ‘care bundle’ search string in this scoping review. This search string was developed by an information retrieval specialist and subjected to peer review by a topic expert. The final search strategy for this scoping review was subsequently subjected to independent peer-review by a university subject librarian. The search terms, and their combinations using the Boolean operands ‘OR’ and ‘AND’ for each database, including the number of returns, are presented in Supplementary File 2 ( https://osf.io/hp5vq/).

Grey literature searching was used to supplement the electronic database searches. OpenGrey System for Information on Grey Literature in Europe, the Open University dedicated grey literature site, the World Health Organization’s International Clinical Trials Registry Platform, and the LENUS repository were searched. Additionally, professional body websites were searched for potential guidelines, policies, or information on the use of care bundles in maternity care. Supplementary File 3 ( https://osf.io/hp5vq/) presents the professional body websites that were searched, and the number of returns retrieved from each of these. Lastly, the reference lists of included records were screened for potentially relevant additional studies not already captured in the searches of the other sources. 

***Study selection*.** Records retrieved from the search were uploaded to EndNote v20 (Clarivate Analytics, PA, USA) initially and duplicates were removed. The remaining records were then uploaded to Covidence (Veritas Health Innovation, Melbourne, Australia) in preparation for screening and selection. To ensure congruency in the interpretation of the scoping review’s eligibility criteria, pilot screening was undertaken on the first 10 records in Covidence by two reviewers independently. Agreement on ‘include’ or ‘exclude’ for these records was 100%. This reassured the review team that both screeners were interpreting the eligibility criteria in the same way, and accurately. Following the pilot screening, the remaining retrieved records were screened by title and abstract by the same two reviewers independently. Records assessed as potentially eligible, or those where relevance was uncertain, were forwarded for full text review. Records at full text were further assessed against the scoping review’s inclusion criteria by the same two reviewers independently. The numbers of, and reasons for, excluding records at full text screening were documented and reported. Any disagreements and uncertainties that arose between the two reviewers at each stage of the screening process were resolved through discussion and consensus. Where consensus could not be reached, a third reviewer would have been consulted, however, this was not required.

### Data extraction

A data extraction tool (DET) developed by the reviewers for the purposes of this scoping review was used to extract the data from the included records (Supplementary File 4: https://osf.io/hp5vq/). The DET was designed using the JBI data extraction template ^18^, piloted on three included records by two reviewers independently and refined accordingly. Three refinements to the original DET (see protocol ^17^) were implemented following the pilot. These were adding rows to extract i) the aim of the study, ii) the authors’ general conclusions and iii) author conflict of interest or disclosure statements. Data were extracted from the included records by two reviewers and then checked for consistency by a third reviewer to ensure depth and completeness of detail; for example, that the full reference details were documented on the DET, or that all components of the care bundle were accurately extracted. As the aim of the scoping review was to identify and describe the existence and type of care bundles, and in line with JBI guidance on scoping reviews ^18^, a methodological quality appraisal of the included records was not performed.

### Data charting and presentation

Data mapping, charting and presentation of the results was achieved by using narrative summaries, Tables, and illustrative Figures. The results of the review are presented sequentially by each of the review’s objectives. Findings are narratively described and then illustratively supported by mapping and charting the data using a combination of chart types, for example, bar and pie charts. The elements of the care bundles were mapped to the clinical conditions narratively supported by tables, where relevant, to illustrate any overlap of elements across care bundle records. Although effectiveness, or other outcomes measures, are not usually the focus of scoping reviews, for studies that reported outcome measures, the findings related to these were extracted to the relevant DET. Similarly, for any included studies that reported on women’s views and experiences of a care bundle, the identified themes were extracted to the DET. Extracting this type of information was helpful for determining the potential need for future systematic reviews in addressing *Objective 4*. 

## Results

### Search and selection

**Figure 1.**
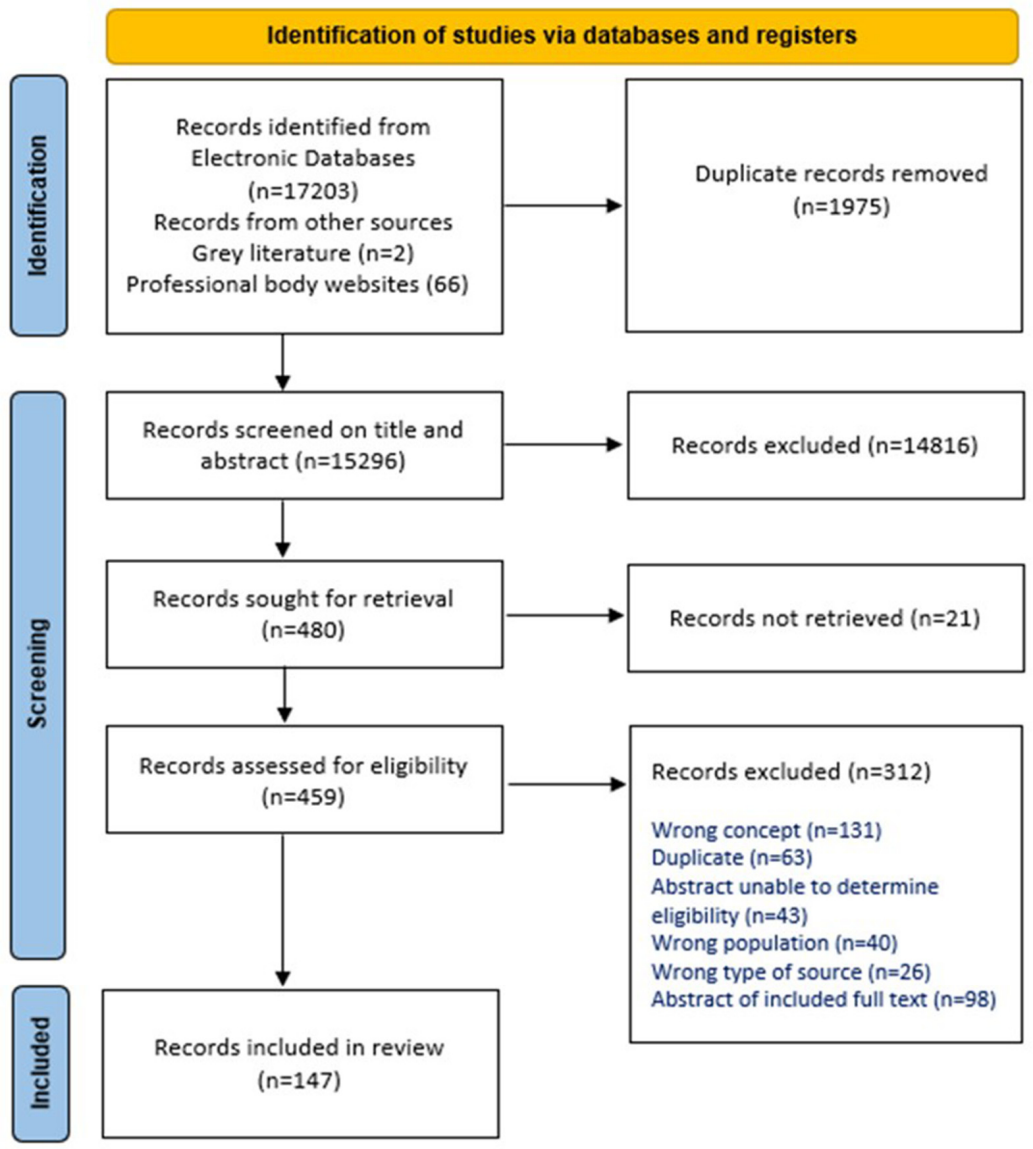
Search & Selection Flow Diagram ^23^.

The search identified 17271 records (17203 from database searching and 68 from other sources). Of these, 1975 were identified as duplicates and were removed. The resulting 15296 records were screened by title and abstract against the review’s inclusion criteria, and 14816 were excluded as they were clearly ineligible. Of the remaining 480 records, we were unable to obtain the full text of 21. This resulted in the retrieval of 459 full text records which were assessed for eligibility. Of these, 312 were excluded as they did not meet the review’s inclusion criteria. The citation details for these 312 records, with exclusion reasons, and the 21 records for which full texts were not retrieved, are provided in an Excel File in the OSF project webpage ( https://osf.io/hp5vq/). In summary, 131 were excluded because they did not report on the review’s concept, for example, a care bundle may have been described or referred to in the record, but the record did not report on the development, evaluation, or implementation of the care bundle explicitly, or the description of the bundle did not meet the IHI definition. A further 63 were identified as duplicates and were removed. Forty-three records were in abstract format only, with insufficient detail in the abstract to determine their eligibility. Forty records were excluded on ‘ *wrong population’*, for example, neonates. This category also included records on care bundles in maternity care where clinicians’ perspectives were sought. The remaining 35 records were excluded as 26 were ineligible types of sources, mainly errata or correction records, and nine were abstracts of already included full text papers and did not offer additional details. No records were excluded for being a non-English publication. One hundred and forty-seven records were ultimately included in this scoping review. Supplementary File 5 ( https://osf.io/hp5vq/) provides the citation details of these records. Figure 1, publicly available for download and use via the Equator Network ( https://www.equator-network.org,/reporting-guidelines/prisma/), illustrates the search and screening process.

### Characteristics of included studies

**Figure 2.**
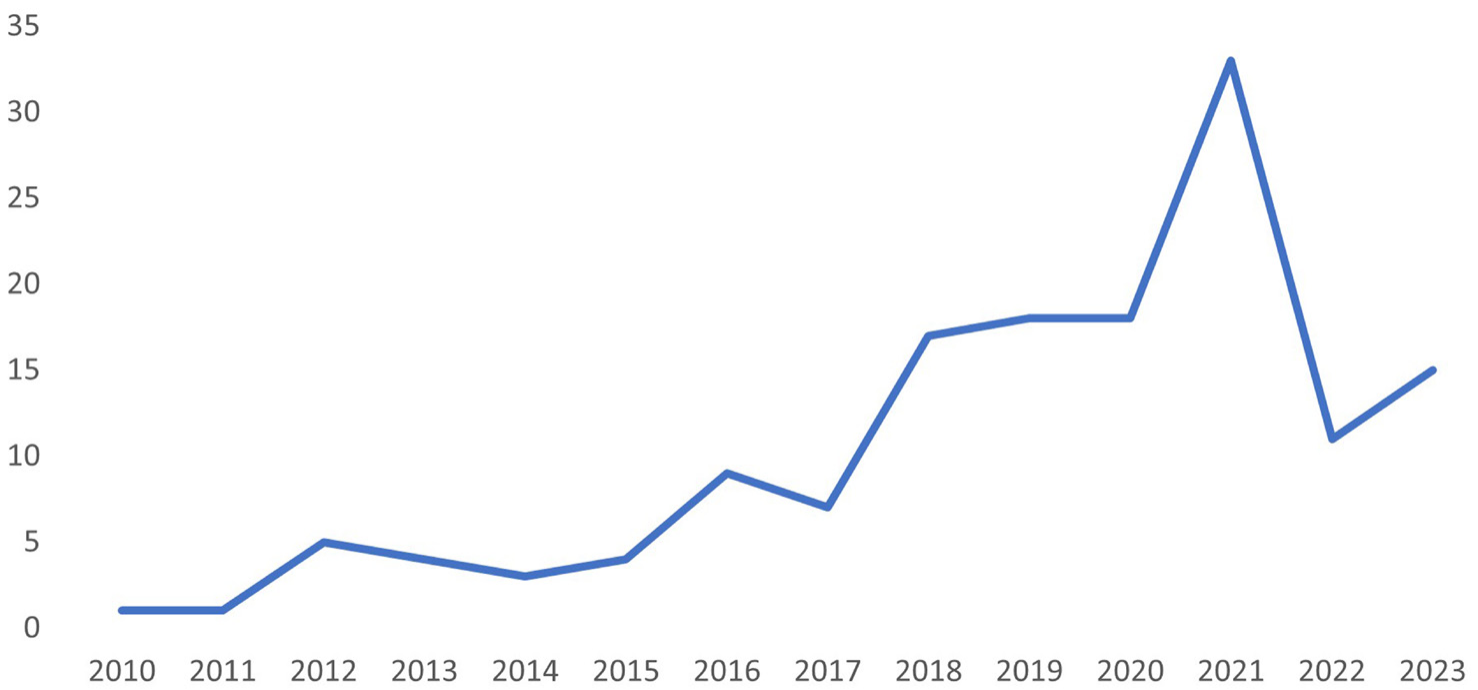
Number of records by year of publication.

Supplementary File 6, Tables S6.1 and S6.2 ( https://osf.io/hp5vq/) provide the detailed summary characteristics of the included records, listed alphabetically by first author. The information presented in Table S6.1 includes the aim, country of origin, study dates (if applicable), type of evidence source, description of participants, type of clinical condition, record category (development, evaluation, implementation), and stage in the maternity continuum (antenatal, labour, birth, postpartum). Additional details, where provided, are presented in Table S6.2 and include details of the study setting, information on the care bundle developer(s), year the care bundle was developed, and disclosures (funding or conflict of interests). Most of the 147 records originated in the USA (n=69), followed by the UK (n=28) and Australia (n=14). Five originated in Canada, three in Africa, three in Pakistan, and a further three involved multiple countries. Two records originated from each of Columbia, Denmark, Egypt, England, Ireland, and Tanzania, and one record from each of India, Italy, Malawi, Nepal, Norway, Scotland, Wales, and Vietnam. For two records, the country of origin was unclear. Figure 2 illustrates the number of records by year of publication. The illustration highlights the incremental expansion of publications in the past 10 years. Most records were published in the last five years (n=95), with 2021 a peak publication year (n=33).

Fifty-five of the included records were in abstract format. The types of evidence sources varied widely with some overlap; for example, 40 were described as before and after studies, utilising both retrospective and prospective approaches. Thirty-two were described as quality improvement projects (QIPs) and 11 as audits, some of which also used before and after designs as well as chart reviews. A further 12 were described as retrospective or prospective observational studies, 10 as randomised trials, three as mixed method studies, three as pilot studies, two as cross-sectional surveys, two as qualitative studies, and one record was a report of an interrupted time series design. A further evidence source category contained a mix of consensus or descriptive development reports (n=18), editorial or opinion pieces on development or effectiveness (n=5), implementation guidance reports (n=2), an evaluation case report (n=1), and a collaborative (n=1) and technical consultation report (n=1). For three records, the type of evidence source was unclear. The study designs of the included records are explored further, by clinical condition, in the *Review Findings* section, sub-section’s *Objective 3* and *Objective 4*.

**Figure 3.**
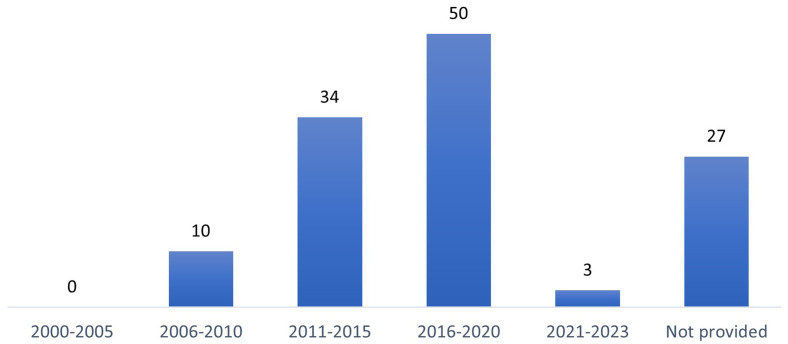
Study commencement dates (in five-year categories).

For 23 included records, a study commencement date was not applicable as the record did not relate to a primary study report. For the remaining 124 records, the study commencement dates spanned from 2007 to 2022, with most studies (n=50) commencing in the five-year category 2016-2020. For 27 study records, the study date was not provided ( Figure 3).

### Objective 1

**Figure 4.**
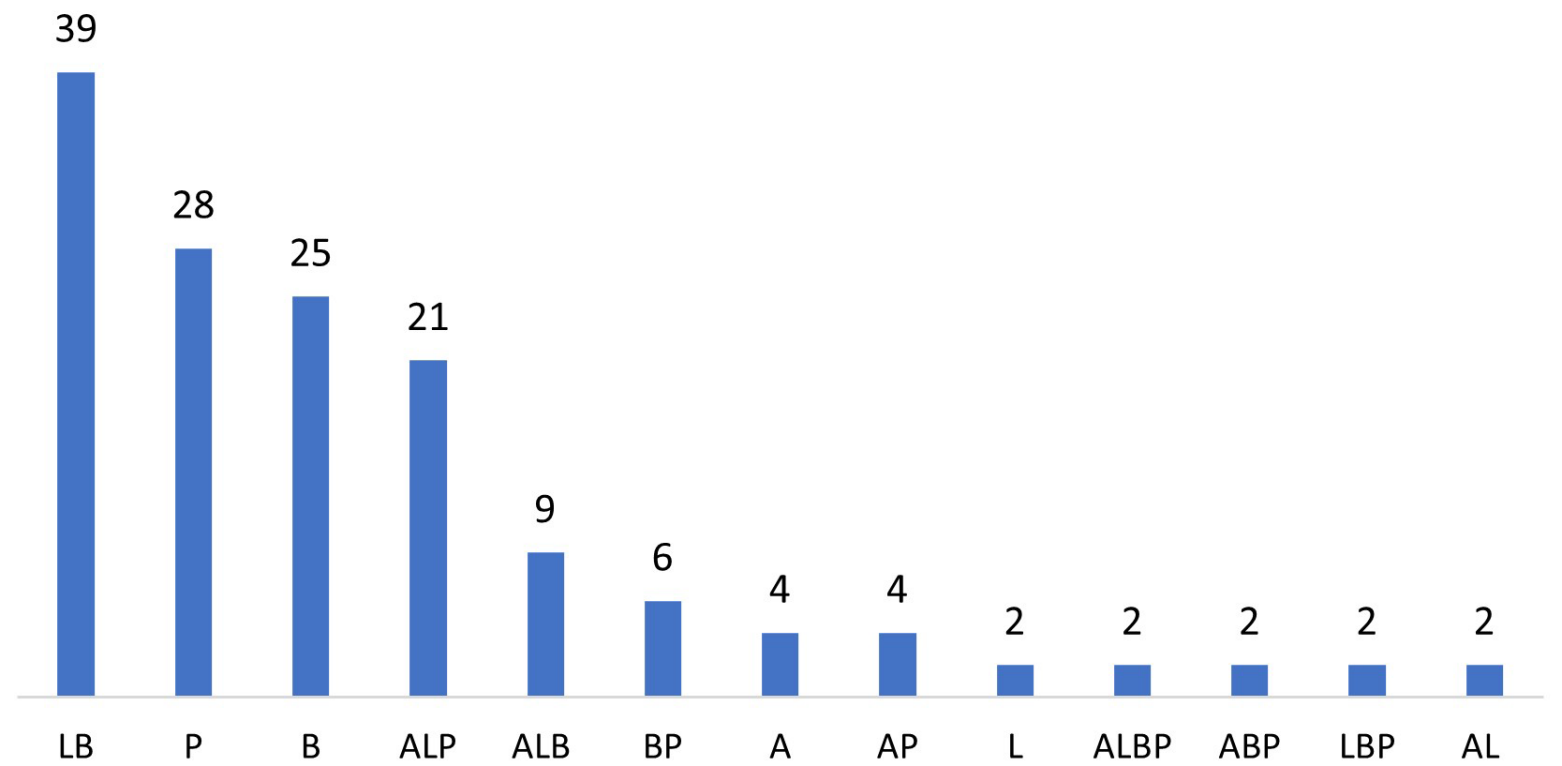
Care bundles by stage in the maternity continuum. Chart Key: A=Antenatal; L=Labor; B=Birth; P=Postpartum, and combinations of these.

Of the 147 included records most reported on care bundle evaluation (n=74). Twenty-four reported on implementation and fourteen on development. The remaining 35 records reported on development, evaluation, and implementation in varying combinations. With regards to the stage in the maternity continuum, most care bundles spanned more than one stage, with care bundles applicable to labour and birth appearing most frequently ( Figure 4).

### Objective 2

Eleven categories of clinical conditions were identified in the 147 records. Twenty-nine, 28, 21, and 14 reported on care bundles for surgical site infection, obstetric haemorrhage, perineal protection, and sepsis, respectively. A further nine reported on care bundles for stillbirth, eight for hypertension, eight for the safe reduction of caesarean section, and three records each for the conditions of enhanced recovery after caesarean, placenta accrete, and perinatal anxiety or depression. A further category, which we referred to as ‘other’, contained 21 records reporting on care bundles for one or two clinical conditions only. These conditions were induction of labour, racial-ethnic disparities (two records), post-caesarean comfort, venous thromboembolism (two records), preterm birth, second stage of labour, catheter associated urinary tract infection, hypotension, safer birth, opioid use, opioid use post-caesarean, severe maternal morbidity, intubation, shoulder dystocia, postpartum care, hypoxic ischemic encephalopathy, hyperemesis gravidarum, birth asphyxia, and fetal growth restriction.

### Objective 3

Supplemental File 7 ( https://osf.io/hp5vq/) presents the bundle elements for the 21 records in the ‘other’ clinical condition category. For the remainder, we present the elements based on the number of records in descending order for each clinical condition.

**Figure 5.**
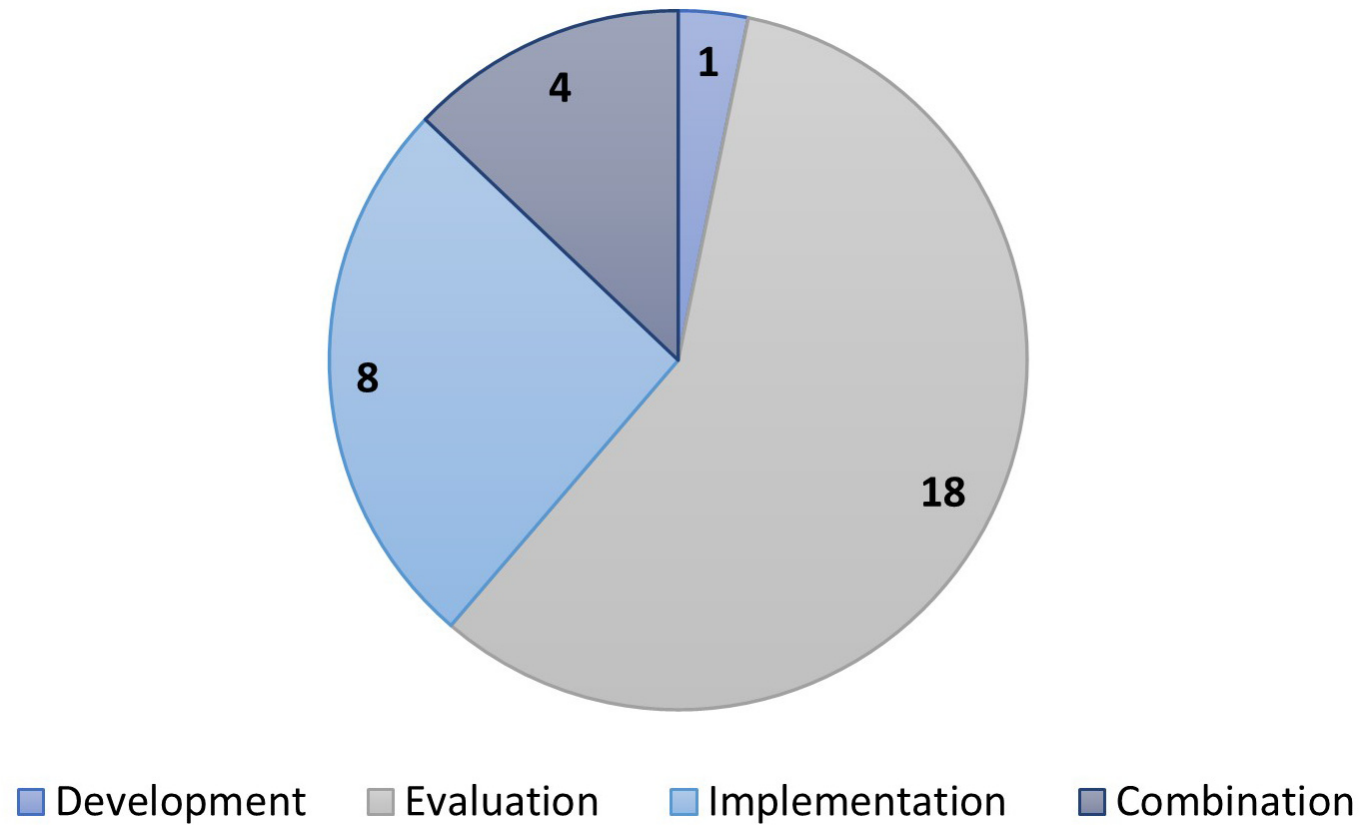
Concept categories for the clinical condition surgical site infection.

***Surgical Site Infection (SSI)*.** Twenty-seven of the 29 records in this clinical category involved women undergoing caesarean section. In the other two, participants were women giving birth to a live baby after 23 weeks gestation, and women at risk for any obstetric related infection. Eighteen records were from the USA, three from Australia, two from the UK, and one each from Canada, Ireland, Norway, and Tanzania. For two records, the country of origin was unclear. Most records were before and after studies, including before and after audits or QIPs (n=21). Five were observational cohort studies, one was a collaborative development process, one was a cross-sectional survey, and for the remaining record, the study design was unclear. Figure 5 presents the concept categories for the 29 records.

The care bundles in the 29 records contained four main element categories which were skin preparation (n=27), prophylactic antibiotic administration (n=22), wound care (n=17), and ‘other’ (n=23). There was some variation in how these elements were applied, however. Prophylactic antibiotic administration, for example, varied in timing from 15 to 60 minutes before surgical incision, to 60 minutes from incision or 60 minutes before skin closure. Similarly, the type and extent of skin preparation varied, ranging from hair removal by clippers rather than razors only to extensive skin preparation, involving hair removal combined with antiseptic skin cleansing the night before or morning of surgery, as well as prior to surgery allowing for 1-2 minutes drying time, and vaginal cleansing. With respect to wound care, some bundles included negative pressure wound dressing (n=7) while others referred to the timing of post-operative dressing removal, the procedure for skin closure, the type of dressing that should be used and education on wound care. Elements in the ‘other’ category included practices such as risk assessment, thermoregulation (intraoperative warming), glove changes during the caesarean procedure, double gloving and removing the placenta by controlled cord traction. Due to the extent of description and variation, details of the elements in the care bundles for SSI are presented in Supplementary File 8, Table S8.1 ( https://osf.io/hp5vq/).

**Figure 6.**
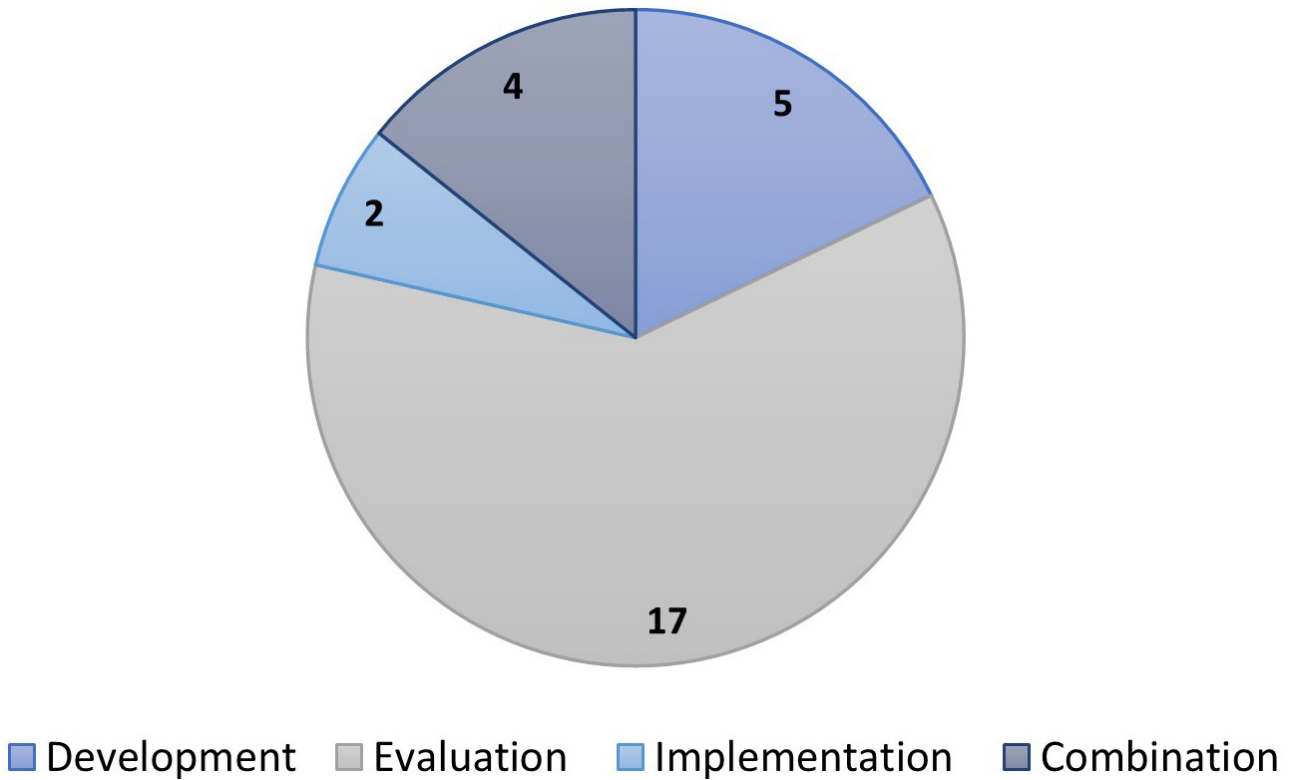
Concept categories for the clinical condition obstetric haemorrhage.

***Obstetric haemorrhage*.** Seventeen of the 28 records reporting on obstetric haemorrhage were concerned with care bundle evaluation ( Figure 6), and most were before and after studies (n=9). Eighteen records were from the USA. Of the remaining 10, three were from Africa, two were international, two were from Columbia, and one record was from each of India, Italy, and Wales.

Although the individual elements in the care bundles were extensive, many of these overlapped across care bundles, while others were included in a few bundles only. Table 1 summarises the elements mapped to the number of records that noted the element in a care bundle. Several records, which may account for some of the overlap, referred specifically to the National Partnership for Maternal Safety (n=5) or the Alliance for Innovation on Maternal Health (AIM) (n=5) Obstetric Haemorrhage Safety Care Bundle.

**Table 1.** Elements in care bundles for obstetric haemorrhage.

Element	Records (n) *
Quantitative blood loss measurement	13
Uterotonic drugs	11
Haemorrhage cart or kit with supplies	11
Response team and/or established management protocols, checklists, or plans	11
Risk assessment	11
Tranexamic acid	10
Team or unit education and/or training, drills, post-drill debriefs	10
Transfusion protocols	9
Multidisciplinary team/Peer review or audit of haemorrhage or near miss	8
Uterine massage	8
Immediate access to haemorrhage medications	7
Intravenous Fluids	6
Active management of the 3 ^rd^ stage labour	6
Support program/debriefs/information for patients, family, and staff	6
Post-event team huddles or debriefs	6
Non-pneumatic anti-shock garment	4
Structured examination and escalation (as needed)	4
Compressive measure (i.e., aortic compression, bimanual uterine compression)	3
Intrauterine balloon tamponade	3
Isotonic crystalloids	2
Point-of-care tests of haemostasis	2
Surgical interventions (e.g., insertion of Bakri Balloon, laparotomy, hysterectomy)	2
Call for Help (OB STAT)	1
Vital signs (defined measurement cycles)	1
Apply oxygen therapy	1

*Three records that referred to the AIM care bundle without listing individual elements are not included in these numbers.

**Figure 7.**
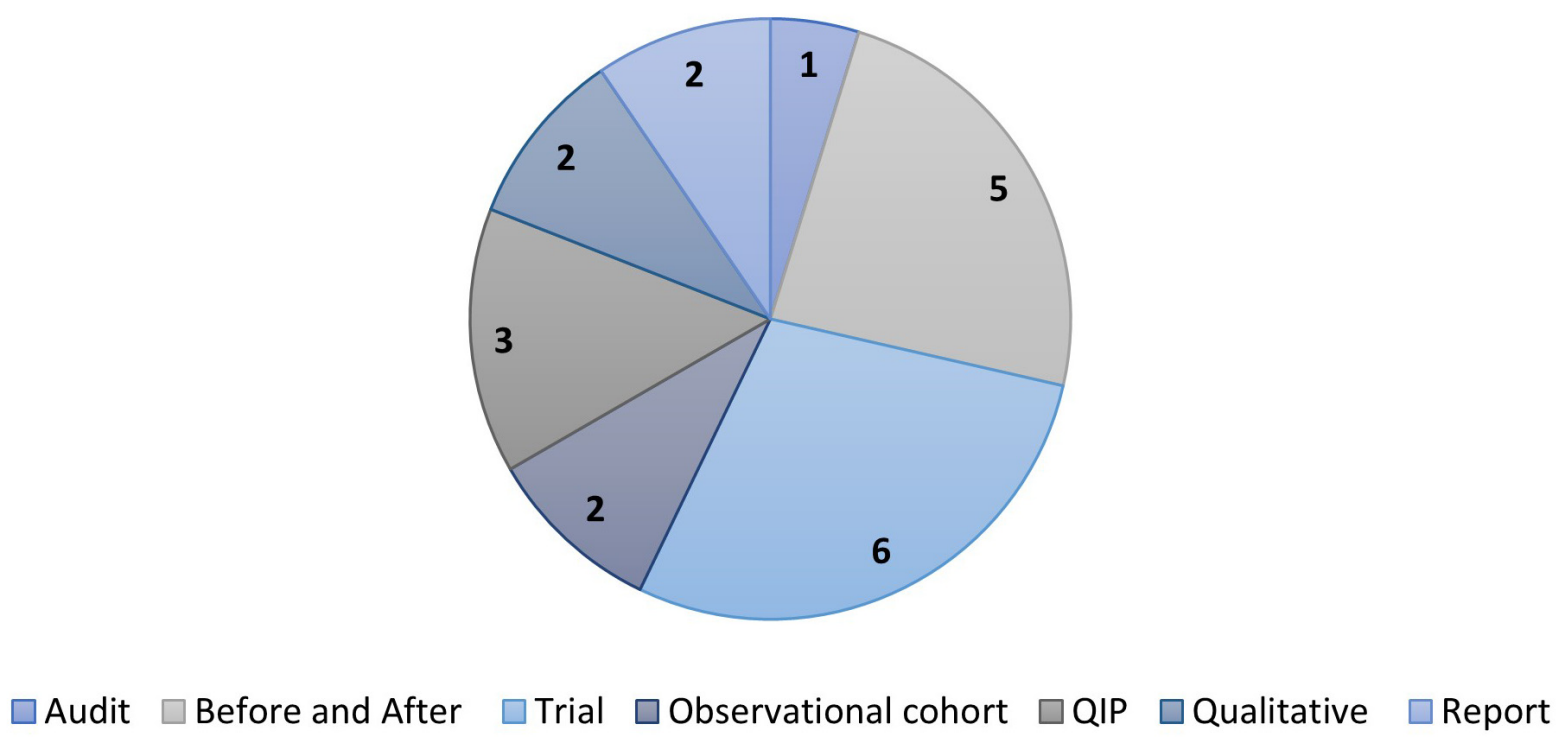
Evidence sources for the clinical condition perineal protection.

***Perineal protection*.** Half of the 21 records on care bundles for perineal protection originated from the UK (n=11). Six were from Australia, two from the USA, and one each from Ireland and Denmark. Sixteen of the records were about evaluation, two implementation and three a combination of these. Six of the 21 records were randomised trial records ( Figure 7). All these records, however, were connected to the same trial; the evaluation of the UK-based OASI Care Bundle ^14^, albeit different aspects (e.g., protocol, effectiveness evaluation, or implementation/process evaluations).

**Figure 8.**
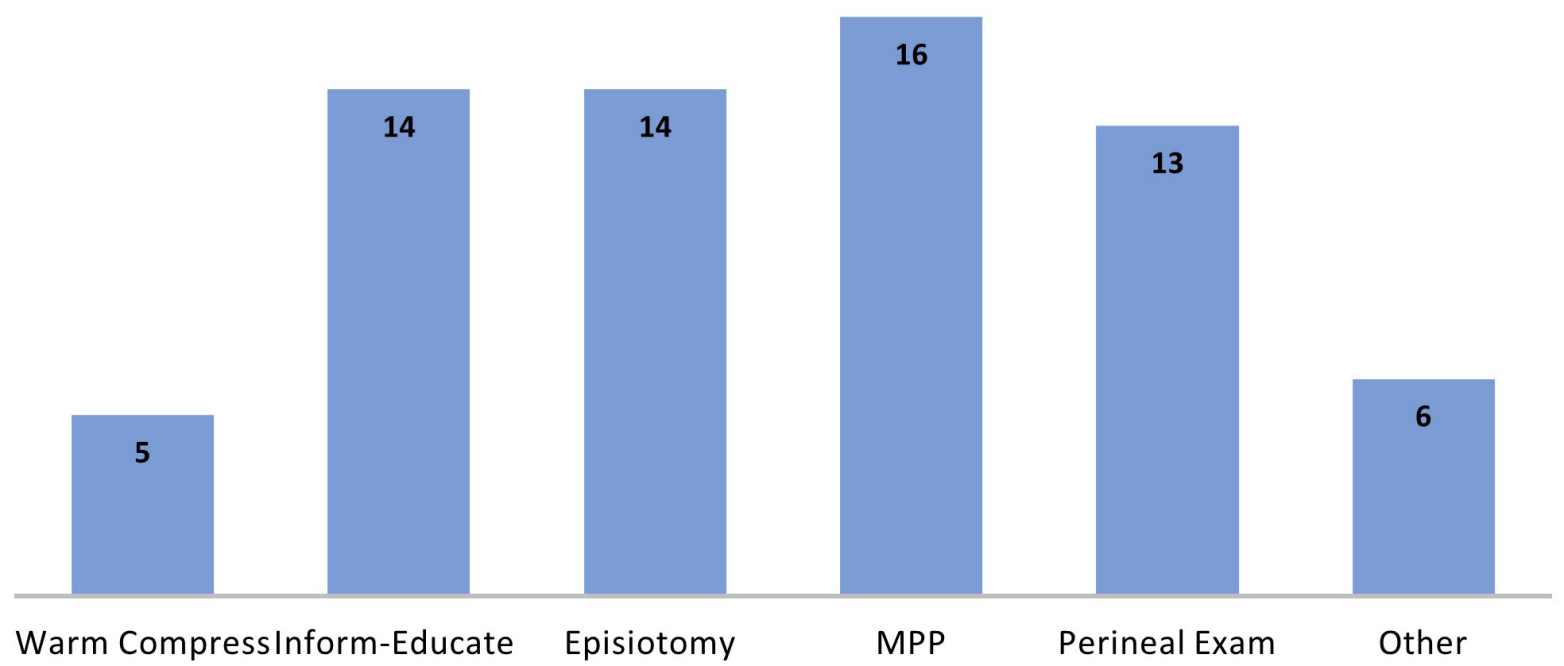
No. of records reporting each element in perineal protection care bundles. MPP: Manual Perineal Protection.

The elements of the care bundles, where sufficiently described, indicated strong overlap across the records, with six main elements identified ( Figure 8). The application of these elements also bore similarity; for example, of the 16 records noting manual perineal protection (MPP), in all cases this meant taking a hands-on approach in supporting the fetal head and perineum during birth. Similarly, the post-birth element of examination, in all bundles, included an examination of the perineum and the rectum. For the element of inform-educate, this centred mainly on the provision of information to women antenatally on OASI and minimising its risk. In some bundles it also included training and education of clinicians in recognition, diagnosis, and surgical repair technique (n=1), retraining in the use of episiotomy (n=1) and communication techniques around the time of birth (n=3). Elements described as ‘other’ included the intervention practices of encouraging upright positioning, changing position every 15 to 20 minutes to help facilitate fetal descent and rotation, verbal coaching to avoid expulsive pushing, and avoiding manual perineal stretching during the second stage of labour. One notable difference in the care bundles, as described in the records, was the element of perineal warm compresses during labour and birth. This was an element in five bundle records from Australia, USA, and Ireland, but not in the OASI Care Bundle ^14^.

**Figure 9.**
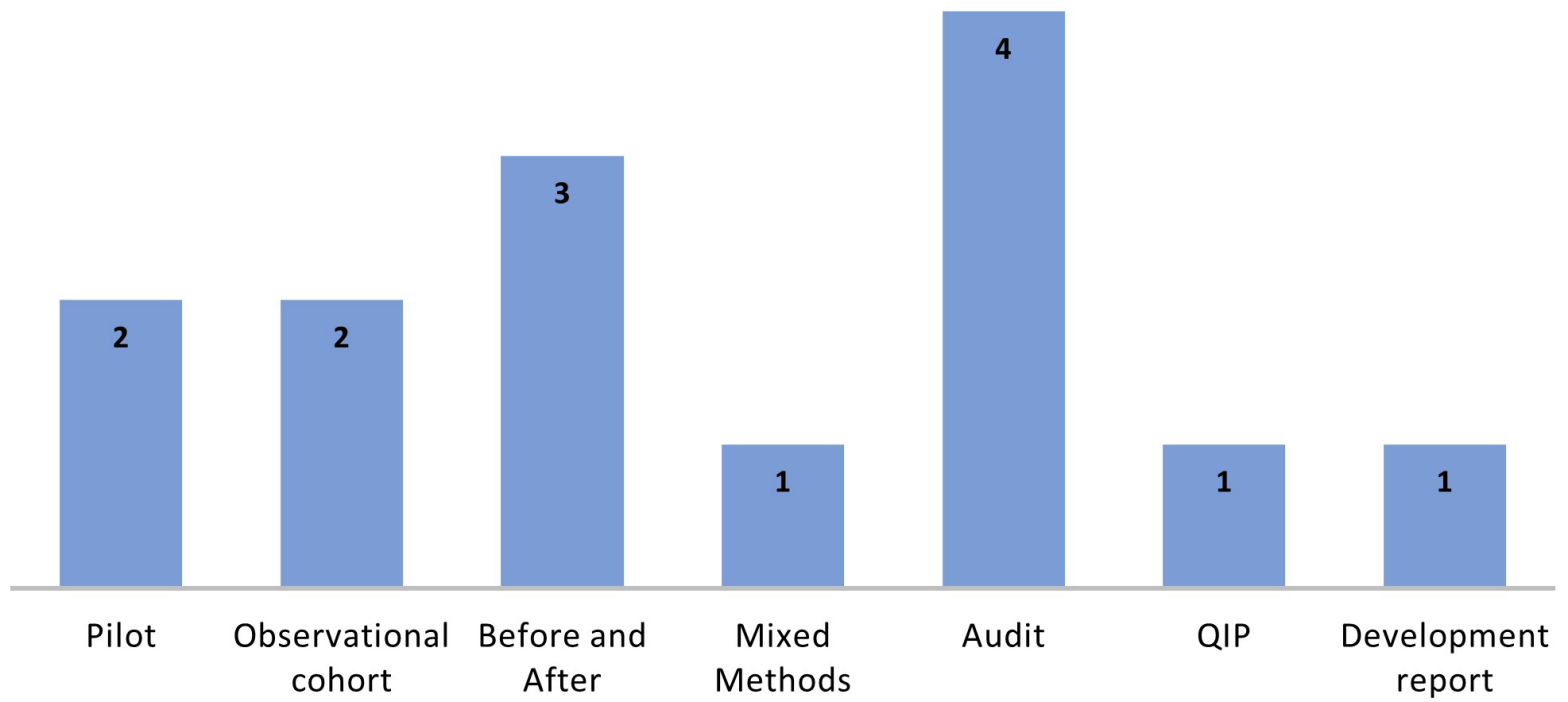
Evidence source type for records on sepsis. QIP: Quality Improvement Project.

***Sepsis*.** Figure 9 illustrates the types of evidence source for the 14 included records on sepsis. Seven records were from the UK, three from Pakistan, two from the USA, and one each from Malawi and multiple countries. Seven records were about implementation, six evaluation and one development.

Six records referred specifically to the Sepsis-Six care bundle, four to the FAST-M (Fluids, Antibiotics, Source identification and control, Transfer (to appropriate higher-level care) and Monitoring) care bundle and one record referred to a Survival Bundle in P-Sepsis (p=puerperal) ( Table 2). All care bundles, as described in the 14 records, included blood cultures, antibiotics, and intravenous fluids as elements. Half of the bundles also included the element of oxygen therapy. Additional elements, some of which overlapped across bundles, also featured. These included urinary output monitoring (n=4), source identification and control, transfer to an appropriate level of care, and ongoing monitoring of mother and neonate (n=4), initiation of vasopressors if indicated (n=2), catheterisation (n=1), fluid balance monitoring (n=1), high vaginal/wound swab cultures (n=1), and consultant arrival in three-hours (n=1).

**Table 2.** Comparison of elements in the Sepsis-Six, FAST-M, and Survival Bundle in P-Sepsis care bundles.

Sepsis-Six Care Bundle *	FAST-M **	Survival Bundle in P-Sepsis
- Oxygen - Cultures - Antibiotics - Fluids - Lactate measurement - Urine output monitoring	- Fluids - Antibiotics - Source identification & control - Transfer to appropriate higher-level care - Monitoring of both mother and neonate as appropriate	- Antibiotics - Cultures - Lactate measurement - Urine output - High vaginal/Wound swab - Consultant arrival in 3hrs (severe sepsis) - Fluid resuscitation

* Sepsis-Six was developed by the UK Sepsis Trust as a set of six elements to be initiated within 1-hour of suspected sepsis by non-specialist front-line healthcare providers. ** Developed for use in low resource settings.

***Stillbirth*.** Of the nine records that reported on stillbirth, five originated in the UK, three in Australia and one in Nepal. The types of evidence sources varied with four reports and one record of each of the following designs: mixed methods study, interrupted time series study, stepped wedge cluster trial, audit, and before and after study. The three records from Australia referred to the Safer Baby Bundle, the five from the UK to the Saving Babies’ Lives Care Bundle, and the bundle described in the record from Nepal was the Scaling Up Safer Bundle. There was considerable overlap with respect to four elements in the Safer Baby Bundle and the Saving Babies’ Lives Care Bundle. Both these bundles included support for smoking cessation, detection/surveillance of fetal growth restriction, awareness and management of reduced fetal movements and intrapartum fetal monitoring. Other elements in the bundles included perinatal audit (n=1), improved awareness of maternal sleep position (n=2), timing of birth for women with risk factors for stillbirth (n=2) and reducing preterm birth through risk assessment (n=2). The Scaling Up Safer Bundle, however, comprised different elements ( Box 2). This might be explained by the wider remit of this bundle which extended to first-day neonatal deaths.

**Box 2. The Scaling Up Safer Bundle**

-    Training and technology improvements

-    Weekly review meetings to track implementation progress and standards

-    Measure quality of care using the Plan-Do-Study-Act approach

-    Improved neonatal resuscitation (using upright bag-masks ^®^, NeoBeat ^®^ newborn heart rate meters, and NeoNatalie live training manikins ^®^, daily skill checks, self-review evaluation checklists)

***Safe reduction of caesarean section*.** Of the eight records in this clinical category, four were from the USA, three from Canada, and one from Australia. Half were concerned with evaluation. The types of evidence source were five before and after studies, a retrospective chart review, a development consensus statement, and an implementation guideline report. Education for clinicians was an element described in six of the eight bundle records. Two of these also included education for women as handouts, videos, and posters. A mix of other elements were included across the bundle records, but these varied ( Table 3).

**Table 3.** Elements in care bundles for the safe reduction of caesarean section.

Element	Records (n)
In-house maternity care provider coverage/expertise	3
Standardization of care specific care practices, guidance or polices	3
Assess individual maternity care provider performance/Audit and Feedback	3
Decision aid	3
Track and report labour and caesarean section	2
Conduct case review	1
Support to facilitate mobility and upright positions	1
Documented management plan at 2hr intervals	1
Supportive one-to-one midwifery care	1
Use of a partograph	1
Use of pre-op checklist	1
Labour walking path	1
Building a culture that supports normal labour	1

***Hypertension*.** Eight records reported on care bundles for hypertension. Of these, two were concerned with evaluation, two with implementation and four with evaluation and implementation combined. All eight records were from the USA, of which two specifically referred to the AIM Severe Hypertension in Pregnancy Bundle and the Hypertension in Pregnancy Safety Bundle. Table 4 presents the elements as described in the remaining six care bundle records.

**Table 4.** Elements in care bundles for Hypertension.

Element	Records (n)
Standard protocols for recognition and/or treatment	5
Clinician education	5
Timely triage	3
Medication access	1
Escalation plan	1
Standard response to maternal early warning score	1
Education of women	4
Support plan for intensive care unit admissions	1
Team communication or review	2
Standardised discharge	1
Follow-up appointments or clinics	2
Dedicated nurse educator	1
Protocols for postpartum hypertensive disorders	1

***Enhanced recovery after caesarean*.** Two records in this clinical category originated from the UK and were described as implementation evaluation QIPs. The third record was from the USA and was an abstract record of a randomised trial. Only one element, early mobilisation, overlapped in all three bundles. Two elements, early return to diet and timely removal of urinary catheter, were included in two bundles. The remaining elements each appeared in one bundle only. These were education for women, postnatal exercises, early return home, prompt discontinuation of intravenous fluids, chewing gum to reduce postoperative ileus, intravenous non-steroidal anti-inflammatories for 24 hours to reduce narcotic use, early removal of dressing, and incentive spirometry encouraged every eight hours.

***Placenta accrete*.** Two of the three records on placenta accrete were evaluation records and the third was an implementation study. The types of evidence source were a case report from Egypt, a pilot study from the UK and a before and after study from the USA. The elements in the care bundles varied, with no overlap across the bundles. One care bundle included three elements, namely, compression of uterine vessels, occlusion of ovarian vessels and transverse compression suture. Another bundle included consultant obstetrician and consultant obstetric anaesthetist presence, blood availability, multi-disciplinary input pre-operative, discussion and consent for interventions, and a level 2 critical care bed. The third bundle included betamethasone administration prior to birth, preoperative balloon catheters, gynaecologic oncology (hysterectomy), cell salvage, vertical skin incision and fundal/high transverse hysterectomy.

***Perinatal anxiety or depression*.** The three records in this clinical category all originated in the USA. Two records were implementation QIPs and the third was a consensus implementation report. The elements in the care bundles minimally overlapped ( Table 5).

**Table 5.** Bundle elements for the clinical condition perinatal anxiety or depression.

Care Bundle 1	Care Bundle 2	Care Bundle 3
- The Action Plan for Depression and Anxiety Around Pregnancy handout - Discussion about the symptoms and help - Lists of mental health resources.	- Mental health screening tools - Protocols for readiness, response, and referral - Education of staff - Responsible individual - Mental health history - Awareness education - Timely support for women - Follow-up from mental health care providers - Multidisciplinary mental health rounds and review of outcomes.	- Perinatal Mood and Anxiety Disorders screening tool and education for women - Referral - Team huddles - Quality improvement implementation.

### Objective 4

Regarding the potential for future systematic reviews, few records reported on randomised trials, rendering the potential for future effectiveness systematic reviews challenging. Of the 10 records that were reports of trials, six of these related to the clinical condition of perineal protection and all were concerned with the OASI Care Bundle. The other trial records also related to one trial on a single clinical condition, for example, a randomised trial of a care bundle for the detection and prevention of obstetric haemorrhage ^24^ and a trial protocol record for the evaluation of the Scaling Up Safer Bundle ^25^. Similarly, although two qualitative studies were identified, both exploring women’s experiences of a perineal protection care bundle ^26, 27^, data are presently limited to meaningfully enable the conduct of a qualitative evidence synthesis. The qualitative studies relate to the UK OASI Care Bundle and an Australian developed perineal protection bundle, both of which for the most part include the same elements. Three prominent themes were identified in each study ( Table 6), with associated sub-themes also described in the study records. Should one or more qualitative studies on women’s experiences of care bundles for perineal protection emerge in the future, a qualitative evidence synthesis could be feasible.

**Table 6.** Prominent themes in qualitative studies of women’s views of perineal protection care bundles.

OASI Care Bundle (UK)	Perineal Care Bundle (Australia)
1. Lack of information and consent to bundle elements 2. Other non-consented and disrespectful treatment 3. Recommendations for hospitals and clinicians	1. Memories of touch 2. Midwife as a supportive guide. 3. Education: women need more information

Systematic reviews of non-randomised studies of care bundles for some clinical conditions may be feasible. Although these types of reviews provide lower-level evidence, as these types of studies are prone to high risk of bias, they could offer evidence on the impact of care bundles in some clinical conditions which could be helpful for clinical decision-makers. For example, for the clinical condition of surgical site infection, 11 included studies provided pre- and post-bundle event and total group dichotomous data. Other included records (n=9) provided proportionate pre- and post-data that might be convertible to event counts or retrievable by contacting study authors. Collectively these data, some of which have already been included in a previous systematic review ^28^, may be suitable for inclusion in a future meta-analysis. In the earlier review, pooled pre- and post-data from 14 studies showed a 3-fold reduction in surgical site infections from 6.2% to 2%. Some of the studies included in the review, however, did not meet the criteria for inclusion in our scoping review because the intervention was not explicitly described as a care bundle ^29– 31^, or it was not clear that it met the IHI definition of a care bundle ^32, 33^. For the clinical condition sepsis, 10 records reported data on compliance to the bundle or to elements in the bundle, offering the possibility of synthesising overall compliant rates. Similarly, 12 records on obstetric haemorrhage provided postpartum haemorrhage outcome data that might be amenable to synthesis. The challenge, however, for any syntheses of data from these non-randomised studies, is homogeneity in care bundle elements across studies. If significant clinical heterogeneity exists in these, this will likely render a meta-analysis of outcome data inappropriate.

## Discussion

This scoping review offers comprehensive insight into the existence of care bundles when caring for women during pregnancy, labour, birth and postpartum. The number of records identified in each clinical category further provide insight into the clinical conditions that care bundles are being designed with surgical site infection, obstetric haemorrhage, perineal protection, and sepsis featuring prominently. Although many care bundles for specific clinical conditions contained several elements that overlapped, there were also many elements that featured in one care bundle only. This may be problematic for standardised care provision across settings. Although standardisation of care in midwifery is widely contested ^34^, in the context of preventing, managing, or treating maternity outcome adversity, having policy, practice, and guideline standards, as well as standardisation of these across settings, is important ^1, 35^. Furthermore, care bundles for specific conditions that contain diverse elements challenges the notion on which care bundles are predicated, that is the aggregation of a discrete set of evidence-based practices. Taking the clinical condition of perineal protection as an illustrative example, the element of perineal warm compresses was included in some bundles (e.g., the Australian developed perineal protection care bundle), but not in others, including the UK-developed OASI bundle. Evidence from systematic reviews ^36, 37^ on the use of warm compresses for perineal protection have demonstrated higher rates of intact perineum in the intervention groups. Although the development of the OASI Care Bundle involved professional body and wider stakeholder involvement ^13^, ignoring an element for which Level 1 Evidence ^38^ exists, is questionable, a view which others share ^39^. A further illustrative example are care bundles designed for stillbirth, some of which include behaviour or lifestyle related interventions (i.e., smoking cessation, improving awareness of reduced fetal movements (RFM), and recommendations around maternal sleep position). Although strong Level 1 evidence exists for the link between smoking and stillbirth ^40^ and RFM and stillbirth ^41^, translating this evidence into an effective intervention can be challenging because it involves behaviour changes. In the large AFFIRM trial (n=409175 women), an intervention designed to increase maternal awareness of RFM and standardise clinical management did not demonstrate a difference in stillbirth rates between the groups ^42^. A systematic review of behaviour-change techniques in the context of stillbirth prevention, however, reported that the Safer Baby Bundle and the Saving Babies Lives care bundle *“…might have had a positive influence in reducing stillbirth rates*” and that this was “ *in concordance with previously developed behaviour change interventions targeting different outcomes, where authors have concluded that multi-layered interventions targeting individual, societal and environmental-level determinants are more effective*” ^43, p.e505^ In this regard, where direct evidence for a discrete care bundle element might be lacking, combining the element with other elements may prove advantageous, emphasising the ‘collective’ intended effect on which care bundles are designed. In the context of stillbirth, however, further evidence is required to support this assertion.

Aside from non-overlap of elements in some care bundles, the development of care bundles with extensive and multi-layered elements is also worthy of discussion. As per the IHI definition, the intention is that care bundles, inclusive of all elements, are applied consistently by all healthcare professionals. To succeed in this, the availability of resources is critical, especially in settings or situations where staff resources might already be stretched ^44^. Furthermore, education and training of staff in care bundle use might also feature prominently as a requirement for implementation, as well as system-wide buy-in and support. For example, with respect to obstetric haemorrhage bundles, of which some contain 10 or more elements ^45, 46^, one study noted that compliance in completing an online module element was >98%, yet compliance with the quantitative blood loss measurement was only 48% ^47^. A further study cited overall bundle compliance of 92%, but compliance with individual bundle elements ranged from 44% to 100% ^48^. Cited barriers to implementation of an obstetric haemorrhage bundle include a lack of leadership support, complexity, lack of resources and poor buy in from staff ^49^. These findings place emphasis on the need for engagement, support and education and training as part of care bundle development, evaluation and implementation.

Although an extensive number of records were identified for inclusion in this scoping review, few records were of randomised trials. This is concerning, as widespread implementation of interventions in the absence of evidence of effect can lead to poorer health outcomes, and once embedded, culturally or otherwise, they can be difficult to de-implement ^50^. The contra-argument here is that care bundles are designed to include elements only for which there is an evidence base. Acknowledging that the before and after studies identified in this scoping review provide important data for evaluation syntheses, the findings of the scoping review point to a need for robust trials on care bundles in maternity care and the need for qualitative studies on women’s experiences and views of these.

### Strengths and limitations

This review provides comprehensive insight into care bundles in maternity care, and for what conditions, offering a valuable resource to clinicians and clinical decision-makers who have interest in this area. The review adhered strictly to methodological guidance for conducting scoping reviews ensuring rigor throughout the process. The search strategy was both specific and sensitive, and our protocol was prospectively published providing transparency in the review process. The availability of many more records on care bundles in pregnancy, labour, birth and postpartum (see OSF Excel File) that were excluded from the review, however, could be viewed as a limitation. Information in these records may have added further value to the scoping review, for example, records on clinicians’ views of care bundles. To maintain scope, however, we had to take the decision to adhere strictly to our participant criterion and to our concept criterion whereby the record must have explicitly reported on development, evaluation, and/or implementation. A further challenge in the conduct of the review, was mapping and charting the data. Although pre-planned in our protocol, the number of records identified for inclusion and the level of data in some of these was unexpected and expansive. To overcome this challenge, we heavily drew on the use of Excel charts and Tables in managing, organising, and presenting the data. Lastly, in collating the elements described in the care bundles, we did not assess the Levels of Evidence ^38^ underpinning these. Although this was not an intention in our review, we recognise that assessing care bundles elements to ensure they meet the requirement of being evidence-based is important. This should be considered in any future effectiveness reviews of care bundles in maternity care.

## Conclusion

This comprehensive scoping review provides insight on the topic of care bundles in maternity care. Interest in care bundles for some clinical conditions, including obstetric haemorrhage, surgical site infection, perineal protection and sepsis appeared prominent. Few studies were found, however, that evaluated the effectiveness of these bundles, and many bundles for similar clinical conditions contained diverse elements. A more global approach to care bundle development, evaluation, and implementation for clinical conditions in pregnancy, labour, birth, and postpartum is required.

## Reporting guidelines

The review is reported as per the Preferred Reporting Items for Systematic Reviews and Meta-Analyses extension for Scoping Reviews (PRISMA-ScR) ^19^ reporting guideline (Supplementary File 1: https://osf.io/hp5vq/).

## Ethics and consent

Ethical approval and consent were not required.

## Data Availability

The data associated with this review were extracted from records retrieved via library or publicly available sources. Extended data are available in Supplementary Files 1–8 in the OSF project page ( https://osf.io/hp5vq/). Data are available under the terms of the *CC-By Attribution 4.0 International* License. The extracted data for all included records is available via a zipped folder titled ‘Data extraction files’ in the OSF project page ( https://osf.io/hp5vq/). If researchers wish to use these data extraction files directly as provided in future projects, the authors of this review must be acknowledged in such cases.
