# Supplementary material for: Care bundles for women during pregnancy, labour/birth, and postpartum: a scoping review
Source: HRB Open Res. 2024 Jun 24;7:40. [Version 1] doi: 10.12688/hrbopenres.13905.1 (PMC11808848; doi:10.12688/hrbopenres.13905.1)
Supplement: Peer review history

# Reviewer report: Reviewer response for version 1

**DOI:** 10.21956/hrbopenres.15250.r43871

**Published:** 2024-12-27

**Authors:** Helena Teede

**Affiliations:** 1 Monash University, Melbourne, Australia

This Scoping review addresses an important issue of evidence based to maternity care and implementation of Care bundles.

 Methodology appears to be robust.

 Whilst the study designs that are accepted, were very broad, they do align well to the aims.

 It is of concern that a substantial number of the source articles were abstract as much of the required data could not be extracted from abstract form.

 Otherwise the extraction and key learnings here appear to be concisely and well presented.

 The discussion could be a little more focused to the appropriate and salient messages included in the conclusion around a more rigorous approach to bundle development and implementation.

 Limitations called capture the nature of the literature, including the abstract predominance and the fact that the scoping review does not include a quality appraisal of the studies.

 Otherwise, I think this is a comprehensive body of work with robust methodology highlighting a key gap in maternity care.

Are the rationale for, and objectives of, the Systematic Review clearly stated?

Yes

Is the statistical analysis and its interpretation appropriate?

Yes

Are sufficient details of the methods and analysis provided to allow replication by others?

Yes

Are the conclusions drawn adequately supported by the results presented in the review?

Yes

Reviewer Expertise:

Pregnancy, implementation science

I confirm that I have read this submission and believe that I have an appropriate level of expertise to confirm that it is of an acceptable scientific standard.

# Reviewer report: Reviewer response for version 1

**DOI:** 10.21956/hrbopenres.15250.r43250

**Published:** 2024-12-12

**Authors:** Soo Downe

**Affiliations:** 1 School of Nursing and Midwifery, University of Central Lancashire, Preston, England, UK

This is a very well designed and comprehensive scoping review, that is reported clearly, with all the essential elements included, and justified.

 Most of the issues that occurred to me as I read the review are addressed in the discussion. These were:

 -That care bundles on the same topic should include the same elements, and that issues arise when either opinion based elements are included, or when there are too many elements, or when level one evidence is not included (as in the OASIS care bundle)

 -The principle that all elements of a care bundle should be applied every time by all practitioners, and that bundles with elements that do not enable this to happen are not effective

 -The tension between this underlying principle of 100% compliance and the right of the woman/childbearing person to decline care; and, fundamentally, the personalisation agenda. This means that care bundles should only be constructed for essential clinical procedures, where the elements included are not contentious, and where they are based on the highest quality evidence of effectiveness.

 My final question was why qualitative views of women/birthing people were included, but not those of clinicians/professionals. I can't see that the justification given is a logical reason for  including the former group but not the latter. However, I assume this will be addressed in any future systematic review.

 In summary, this is an authoritative  paper in an important area of practice. The findings should give pause to those using and developing care bundles in maternity care. They also provide a strong impetus for funders to support evaluation of the intended and unintended consequences of their introduction in terms of effectiveness, efficacy, acceptability, equity, feasibility, and impact on accessibility to maternity care, as a matter of some urgency

Are the rationale for, and objectives of, the Systematic Review clearly stated?

Yes

Is the statistical analysis and its interpretation appropriate?

Not applicable

Are sufficient details of the methods and analysis provided to allow replication by others?

Yes

Are the conclusions drawn adequately supported by the results presented in the review?

Yes

Reviewer Expertise:

Maternity care, organisation of care

I confirm that I have read this submission and believe that I have an appropriate level of expertise to confirm that it is of an acceptable scientific standard.

# Reviewer report: Reviewer response for version 1

**DOI:** 10.21956/hrbopenres.15250.r42936

**Published:** 2024-11-21

**Authors:** Mandie scamell

**Affiliations:** 1 City university london, London, UK

Thank you for giving me this opportunity to review this really interesting article - a scoping review on care bundles in maternity care.  I really enjoyed reading this article and would recommend it for indexing.

 My main concern is that the article is badged as a systematic review but this is not claimed or attempted by the authors.  This is a thorough scoping review which reads well and raises a range of interesting points.

 Other minor feedback points include the following:

 1. The alignment between the objectives and the findings on each was not always clear to me.  I wondered whether the wording could be adjusted to fit what you did more.

 2. I would have found it helpful to have a reminder of what each objective was within the findings. This would avoid having to scroll back up through the article.

 3. There is a sentence in the discussion that almost equates intrapartum care with midwifery care which was a bit of a surprise.   As you say, standardised, evidence based care  is essential for the management of low probability high impact pathology.  However, midwifery care is purportedly about promoting spontaneous physiology (a holistic approach that is mindful of the physiological complexity of labour and birth) in ways that aim to reduce the risk of such pathologies, right?  I would suggest that if the authors of this article are interested in midwifery intrapartum care as well as obstetric intrapartum care (focussed on the management of pathology more than the promotion of physiology) that the absence of any physiologically focussed care bundles is a significant omission warranting comment and discussion.

Are the rationale for, and objectives of, the Systematic Review clearly stated?

Yes

Is the statistical analysis and its interpretation appropriate?

Yes

Are sufficient details of the methods and analysis provided to allow replication by others?

Yes

Are the conclusions drawn adequately supported by the results presented in the review?

Yes

Reviewer Expertise:

intrapartum care, midwifery, risk, bioethics, critical medical anthropology

I confirm that I have read this submission and believe that I have an appropriate level of expertise to confirm that it is of an acceptable scientific standard.
